# An interdisciplinary approach to ensure adherence to exercise and potentially reduce adverse cardiac events in cancer patients: primers in cardio-oncology

**DOI:** 10.3389/fonc.2025.1649742

**Published:** 2025-09-29

**Authors:** Simon Wernhart, Caterina Fiorentini, Bernadine Madl, Louisa Kuehberger, Simon Glowka, Elena Murr, Sabine Grill, Marion Kiechle, Hana Alguel, Florian Bassermann, Stephanie E. Combs, Juergen E. Gschwend, Mark J. Haykowsky, Martin Halle

**Affiliations:** ^1^ Department for Preventive Sports Medicine and Sports Cardiology, TUM School of Medicine and Health, TUM University Hospital, Technical University of Munich (TUM), Munich, Germany; ^2^ DZHK (German Centre for Cardiovascular Research), Partner Site Munich Heart Alliance, Munich, Germany; ^3^ Department of Gynecology and Obstetrics, Technical University Munich (TUM), Munich, Germany; ^4^ Comprehensive Cancer Center, Technical University Munich (TUM), Munich, Germany; ^5^ Department of Hematology and Oncology, Technical University Munich (TUM), Munich, Germany; ^6^ Department of Radio Oncology and Radiation Therapy, Technical University Munich (TUM), Munich, Germany; ^7^ Department of Urology, Technical University Munich (TUM), Munich, Germany; ^8^ Integrated Cardiovascular Exercise Physiology and Rehabilitation Lab, College of Health Sciences, University of Alberta, Edmonton, AB, Canada; ^9^ Hochgebirgsklinik, Davos, Switzerland

**Keywords:** cardio-oncology, exercise testing, cardiotoxicity, cancer therapy-related cardiac dysfunction, long-term adherence

## Abstract

Exercise training can improve mortality, quality of life, exercise capacity and morbidity in cancer patients. Although current cardio-oncological guidelines recommend exercise intervention in cancer patients, a structured, interdisciplinary approach from diagnosis to maintenance of therapy including repetitive sports cardiological assessment to guide exercise intervention has not been established. Currently, exercise prescriptions are based on assessment of peak oxygen consumption, which does not differentiate between central (stroke volume and heart rate reserve) and peripheral (peripheral oxygen difference) limitations. Knowledge of these mechanisms could facilitate more effective exercise prescriptions because cancer subtypes may respond differently to exercise stimuli requiring individualized and cancer-specific exercise intervention. Our approach uses simultaneous cardiopulmonary exercise testing and stress echocardiography to analyze the entire oxygen cascade in one exam. Based on these findings, we propose individualized assessment of treatment and cardiovascular risk as well as exercise prescriptions. As cardiopulmonary limitations indicative of cancer therapy-related cardiac dysfunction or cardiotoxicity may first be detected during exercise, our approach may help to address cancer therapy-related adverse events earlier. Our approach contains repetitive exercise testing which should serve to re-assess efficacy of exercise intervention. Results of each sports cardio-oncological assessment is fed back to the treating oncologists as it may provide valuable insights to adapt treatment regimens. In addition, we propose a transition from supervised on-site and home-based to self-directed training, which may achieve better long-term training adherence from prehabilitation to post-treatment. In the advent of precision medicine and oncology we provide a concept for precision sports cardio-oncological care to tailor individual exercise prescriptions based on pathophysiological findings during exercise testing.

## 1 Introduction

Cardiorespiratory fitness (CRF), measured by peak oxygen consumption (VO_2peak_), is a major determinant of cancer-associated morbidity and cardiovascular events in cancer patients (1). An improvement of 1 metabolic equivalent (MET, which equals 3.5 ml/kg/min) of exercise performance has been associated with a 10–25% relative risk reduction in all-cause and cardiovascular mortality (2), while low CRF has been associated with poor quality of life, reduced cardiac performance during exercise, a worse cardiovascular risk profile, and higher morbidity in cancer patients (3, 4). Adjunct exercise training has shown positive effects on clinical and functional status, therapeutic effects of anticancer treatment (5), cancer recurrence, mortality and morbidity as well as a reduction in cardiovascular risk factors and events in cancer (4, 6–9), leading to a reduced risk of premature mortality (9–11). However, in the absence of exercise intervention CRF remains severely reduced during and after termination of cancer therapy (12–14). Thus, there is a pressing need to early counteract this decline by establishing exercise programs in the prehabilitation (15), neoadjuvant (16, 17), and adjuvant phases (18, 19) and maintain its effects by providing long-term concepts for outpatient sports cardio-oncological care. These programs should not be limited to localized or regional cancer but should also include metastatic disease (20). Although there is agreement on the positive effects of aerobic and/or resistance training in cancer patients, the effect size varies, which may be due to the different exercise intervention regimens of local facilities and cancer-specific response to exercise (16, 18, 21–25). The wide-spread introduction of cardiotoxic cancer treatment leading to cancer therapy-related cardiac dysfunction (CTRCD) (26), defined by a deterioration of cardiac biomarkers, resting left ventricular ejection fraction and/or global longitudinal strain, necessitates early involvement of cardiologists.

### 1.1 Gaps of current knowledge on exercise training in cancer patients

Although current cardio-oncological guidelines (27) recommend exercise training in cancer patients, there are no specific concepts to ensure sustained patient care from diagnosis to long-term follow-up. In addition, cooperation between medical disciplines, for instance oncology, gynecology, urology, hematology, gastroenterology, cardiology, medical and radiation oncology and para-medical disciplines such as physiotherapy, sport sciences and nutrition, is often complicated by a lack in common infrastructure. Furthermore, sports scientists are required to tailor exercise programs to the patients´ needs and individual response to cancer therapy (28). In addition, a lack of studies combining nutritional support and training has been lamented on (29), which also emphasizes the need for cooperation between an “exercise team” and “nutrition team” to balance energy expenditure and demand (29, 30). Integration of all these sub-disciplines under guidance and coordination of the treating oncological specialist is a herculean task but is necessary to stabilize or even improve all components of physical performance and quality of life during different phases of cancer treatment. We provide a concept of interdisciplinary cooperation through different treatment phases of a cancer patient.

Improvement of VO_2peak_ through exercise training is essential for prognosis but provides too little information on the physiological impact of exercise training. Determinants of VO_2peak_ include cardiac [heart rate and stroke volume reserve (12, 31)] and peripheral muscular adaptations [arteriovenous oxygen difference (31, 32)], both of which can be improved through exercise in a specific cohort of cancer patients (18). However, current exercise oncology regimens do not take into account baseline (before treatment initiation) assessment of VO_2peak_ determinants to tailor individual exercise programs (33) as exercise response may differ depending on the molecular cancer signature, patient characteristics and treatment phase. In the advent of targeted therapy and precision oncology, the implementation of precision sports cardio-oncology seems to be a logical and necessary next step to further improve clinical outcome and survival beyond cancer-specific therapy. Assessment of VO_2peak_ determinants during exercise testing can be achieved by the gold standard invasive right heart catheterization or exercise magnetic resonance imaging, which are neither feasible during daily routine, nor cost-effective (18, 33). A feasible approach in sports cardiology is simultaneous cardiopulmonary exercise testing (CPET) with capillary blood gas analysis from the ear lobe and stress echocardiography. This approach can determine stroke volume increase, heart rate development and arteriovenous oxygen difference in one exam. Physicians may be able to reveal early signs of cardiotoxicity and cancer therapy-related cardiac dysfunction (e.g. exercise-induced decline of left ventricular ejection fraction or global longitudinal strain), which may not be manifest during resting echocardiography. We present a new approach as a clinically feasible way to analyze the entire oxygen cascade in one exam during sports cardio-oncological routine, which provides a deeper insight into exercise pathophysiology of cancer patients than established approaches and provides the basis for tailored, individualized exercise prescriptions.

Long-term adherence to exercise is a major concern in cancer patients. This is also due to limited availability of rehabilitation and training facilities for on-site training and a lack of transition protocols from supervised on-site training to home-based or telemedicine-supported offers. We provide a proposal to accomplish this transition in sports cardio-oncological care.

Contemporary cancer therapy has led to a reduction of cardiotoxicity and CTRCD by applying reduced dosage of cardiotoxic chemotherapy, more efficient and targeted therapy as well as advanced cardiac-sparing radiation techniques (e.g. respiratory motion management) (27, 34). Our approach of repetitive, simultaneous CPET and stress echocardiography stresses the potential of early detection of cardiotoxicity and CTRCD. Our proposal of exercise intervention and repetitive adaptation of exercise prescriptions may have the potential to further reduce CTRCD and cardiotoxicity on top of state-of-the-art cancer therapy.

## 2 Methods

### 2.1 A holistic approach to facilitate long-term care for cancer patients

We propose an interdisciplinary approach which is being launched at our university medical school facility to facilitate long-term holistic care of patients diagnosed with cancer. The aim is to ensure long-term adherence to lifestyle modifications and exercise training, which has been shown to be a major obstacle for preservation of health in patients after successful cancer treatment (35). This effect of waning of CRF after leaving a structured, on-site exercise program has also been observed in patients with heart failure with preserved ejection fraction (36). In addition, exercise trials in cancer and heart failure patients have demonstrated that adherence to exercise training is key to VO_2peak_ improvement (18, 37).

**Figure 1:**
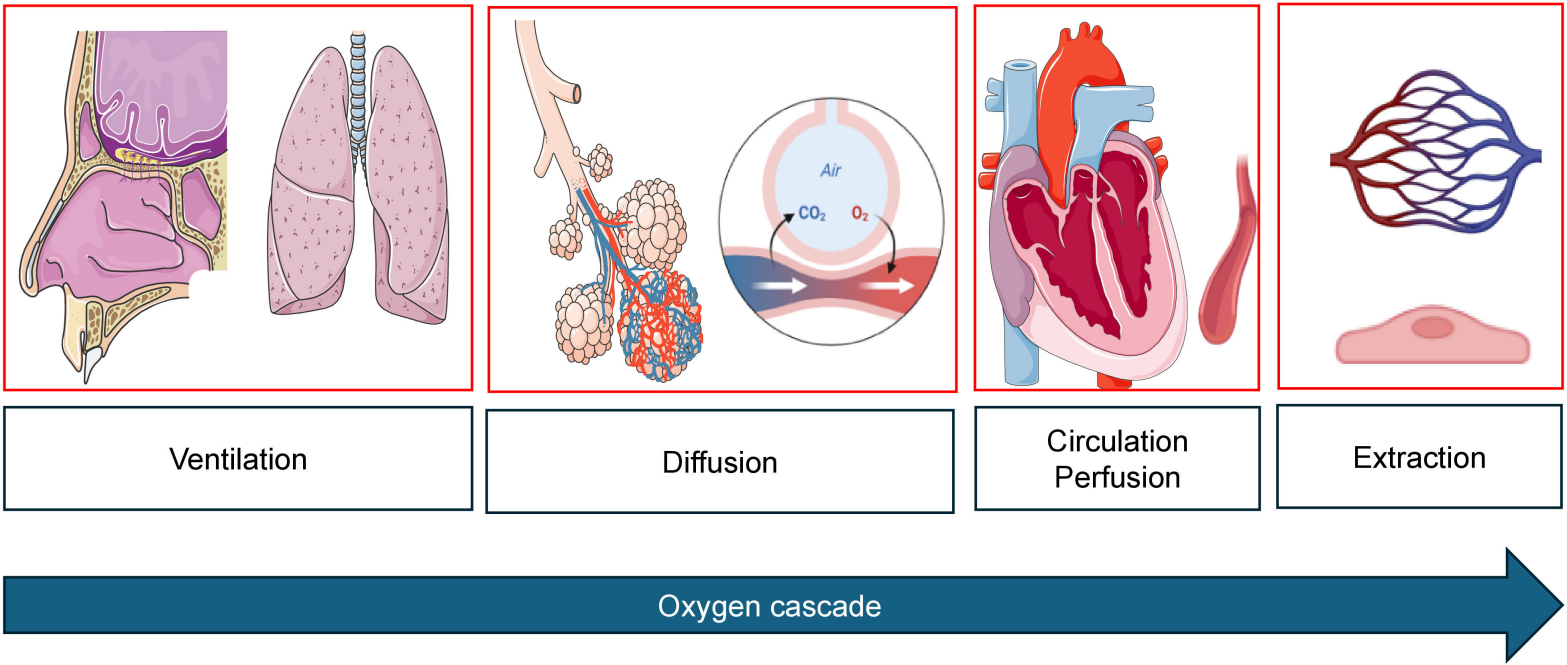
Oxygen cascade from mouth-to-mitochondrion. During cancer therapy, impairment can occur at any level. Images created with biorender.com and smart.servier.com.

Our “cancer team” consists of medical oncologists, radiation oncologists, urologists, gastroenterologists, hematologists, cardiologists, sports scientists, psychologists, physiotherapists, and nutrition experts who repetitively meet in a board-like fashion not only to schedule medical and radiation cancer therapy, but to integrate exercise and nutrition into the treatment algorithms from the prehabilitation to the neoadjuvant, adjuvant and long-term maintenance phase (**Figure 1**).

**Figure 2:**
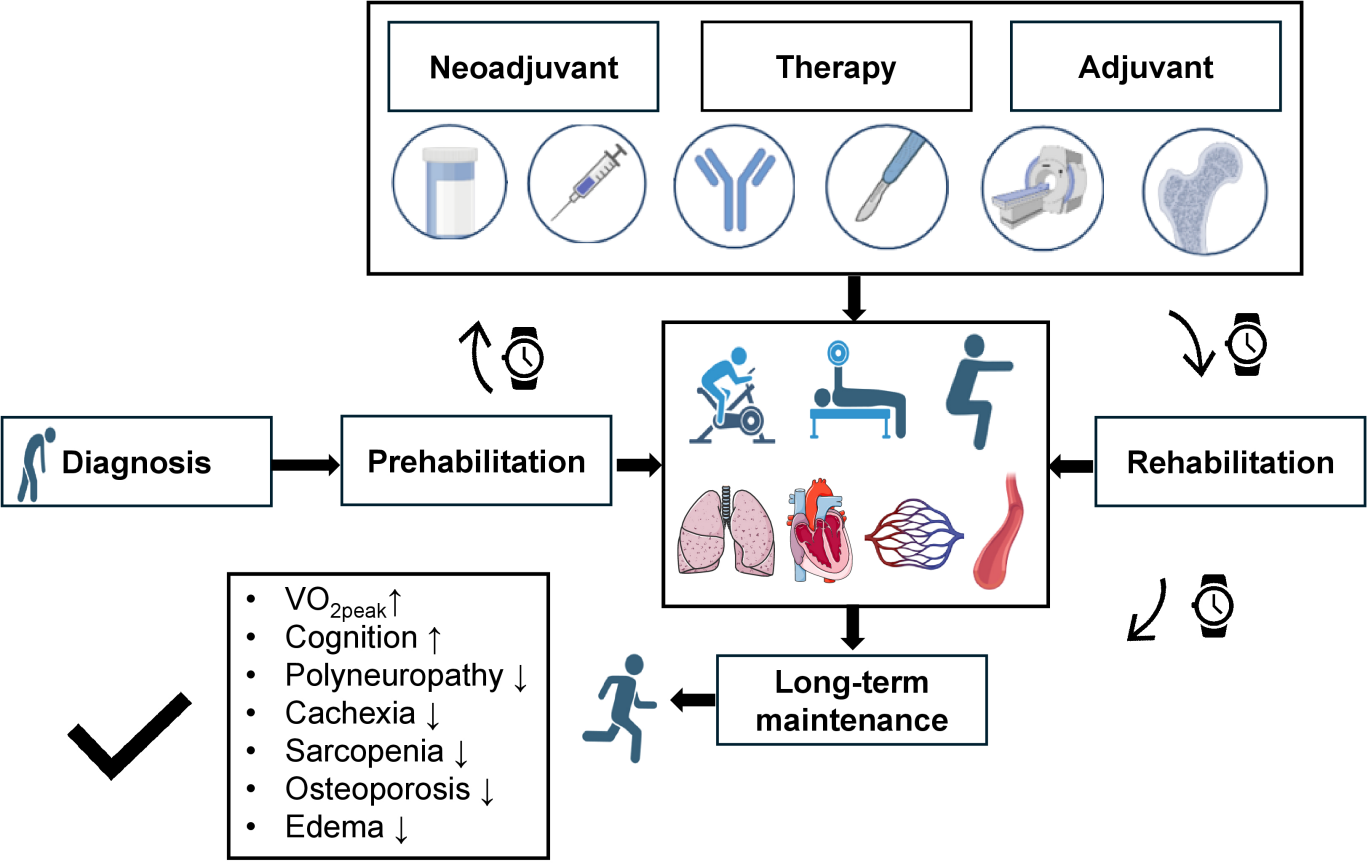
Concept of long-term sports intervention during the phases of cancer treatment. Upon cancer diagnosis prehabilitation is started to facilitate response to cancer treatment following initial sports cardiological assessment. Reduction of exercise capacity can be caused by pulmonary, cardiac, circulatory, and peripheral limitations, which can all be positively influenced by exercise training. Exercise training always integrates aspects of endurance, resistance and balance training. During neoadjuvant and adjuvant therapy (consisting of hormone-, chemo-, and immunotherapy as well as surgery and radiation therapy; the process of bone marrow transplantation is also supported by exercise intervention) exercise training is continued in an in- and outpatient setting. By induction of maintenance therapy patients enter the rehabilitation phase. Here, continued sports cardiological follow-ups are performed to ensure adherence to long-term exercise training. All these measures should lead to preservation and even improvement of peak oxygen consumption (VO_2peak_), increase cognition and reduce cachexia, sarcopenia, osteopenia, polyneuropathy and peripheral edema. Images created with biorender.com and smart.servier.com. VO_2peak_: Peak oxygen consumption.

Sports cardiological surveillance includes repetitive testing of cardiac biomarkers, echocardiographic assessment of cardiac function at rest to detect early signs of new onset cardiovascular risk factors, CTRCD, cardiotoxicity and heart failure. CPET is used to determine exercise training intensity levels in the individual treatment phase and provide objective measures of progress. However, CPET, used simultaneously with stress echocardiography to analyze wall motion abnormalities, stroke volume reserve during exercise, dynamic valve pathologies, or signs of exercise-induced pulmonary hypertension (e.g. triggered by tyrosine kinase inhibitors), also elucidates cardiopulmonary limitations which warrant further clinical investigation before cancer therapy can be continued. By combining CPET (including flow-volume curves to assess hyperinflation and dynamic airway obstruction), blood gas analysis and stress echocardiography, the oxygen cascade can be analyzed entirely in a time- and cost-efficient way (**Figure 2**). This combined approach gaining individual real-time information on the entire oxygen cascade in one exam has not been established in exercise oncology. Early cancer or therapy-induced impairments can thus be detected.

**Figure 3:**
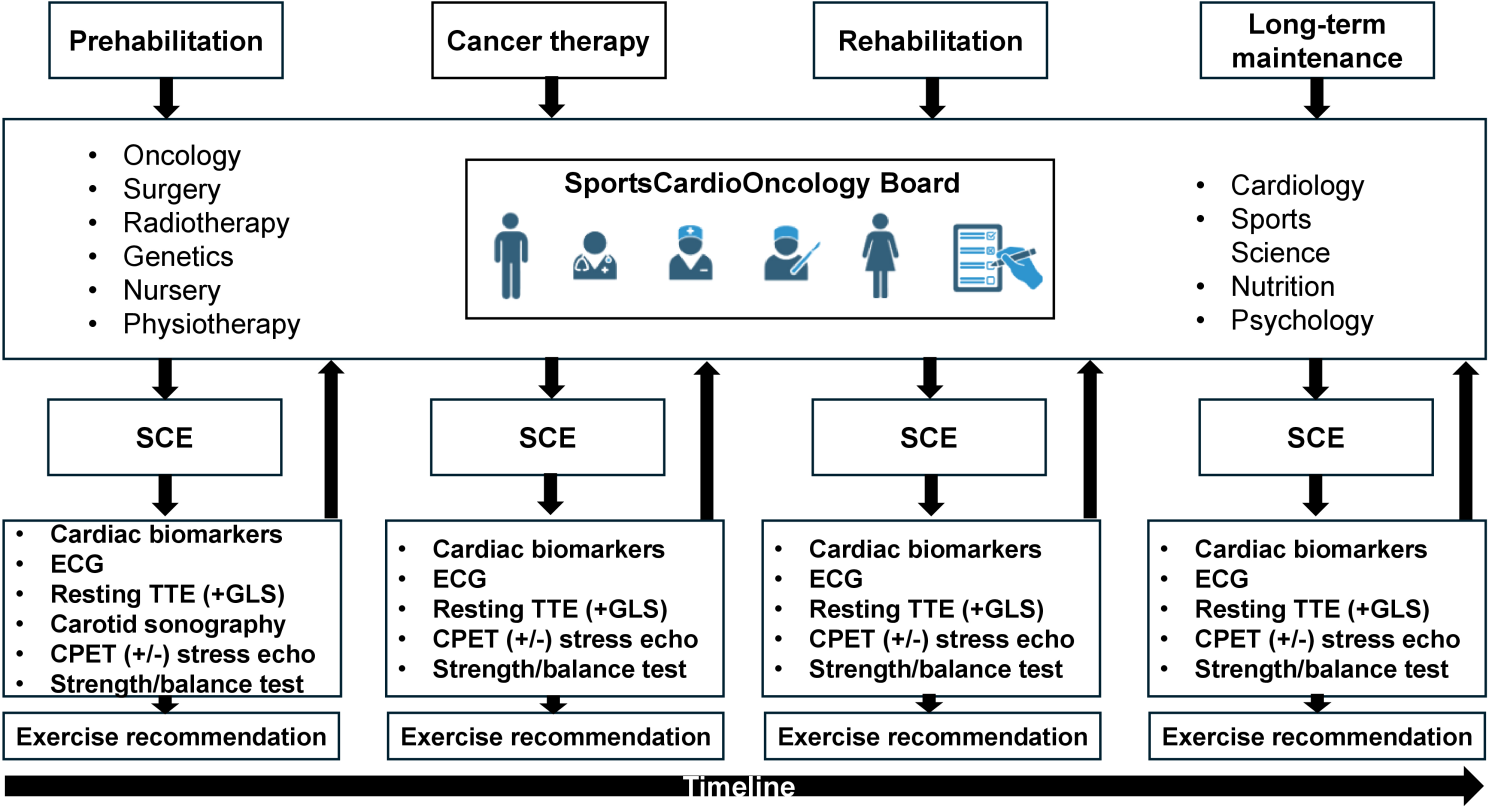
Workflow of the SportsCardioOncology Board. In all phases patients are discussed in the interdisciplinary board, which consists of several medical and paramedical disciplines. A sports-cardiological examination (SCE) is performed after every board meeting and results are reported back to the board (arrows). SCE consists of a cardiological baseline examination of cardiac biomarkers and, if not previously available, measurement of a lipid panel and glucose. A blood pressure profile is performed at the initial presentation according to current guidelines on hypertension (38). Resting echocardiography routinely assesses global longitudinal strain (GLS). At first visit, carotid sonography aims to reveal the plaque burden. Cardiopulmonary exercise testing (CPET) including flow-volume curves and blood gas analysis aims to delineate exercise-induced limitations. If indicated, simultaneous stress echocardiography is added to analyze wall motion abnormalities, signs of pulmonary hypertension and dynamic valve pathologies. Based on these findings tailored exercise recommendations are provided. SCE also includes testing of strength, balance and overall physical performance tests. CPET, Cardiopulmonary exercise testing; GLS, Global longitudinal strain; SCE, Sports-cardiological examination. Images created with biorender.com.

Findings of initial and follow-up sports cardiological examinations (SCE), which are performed in every phase, are discussed on an interdisciplinary board (*SportsCardioOncology Board*), including oncological specialists (hematology, gastroenterology, gynecology, urology, medical and radiation oncologist), cardiologists, sports scientists, and experts from genetics, nursing, psychology and nutrition (**Figure 3**). If contraindications for exercise training are detected, further diagnostics (e.g. left/right heart catheterization, magnetic resonance imaging) are initiated (27, 39).

### 2.2 Individual contributions in the cancer team

Each member of the “cancer team” is informed on the baseline sports cardio-oncological assessment. Results are presented by the sports cardiologist and sports scientist. A proposal for an individualized exercise program for endurance, resistance and balance training is provided by the sports scientist and physiotherapists and a selection of exercises is displayed. The medical and radiation oncologist as well as an oncological surgeon comment on the feasibility of the exercises from a medical perspective. This is important because during surgery or radiation therapy muscles involved in the field of radiation or surgery may be more vulnerable to injuries. Feasibility of each individual exercise is discussed among these disciplines. Especially in patients with prostate cancer and bone metastases, discussion of feasibility of resistance training with medical and radiation oncologists as well as surgeons is essential to deliver the beneficial effects of exercise and prevent injuries.

Psychologists also comment on the workload of exercise. Exercise interventions are also given to cancer patients receiving palliative treatment who sometimes also need psychological support. Psychologists discuss whether the proposed exercise workload could induce excessive psychological stress in the patient.

Nutrition experts design recommendations based on the prescribed exercise intervention. This is of particular importance since caloric turnover needs to consider the amount of exercise. Resistance training is an essential part of exercise in cancer patients to mitigate the effects of cachexia and sarcopenia. Supplementation of macronutrients (especially amino acids) is calculated by considering the exercise workload.

### 2.3 Implementation of repetitive re-assessment and exercise prescription from pre-to rehabilitation

**Figure 4:**
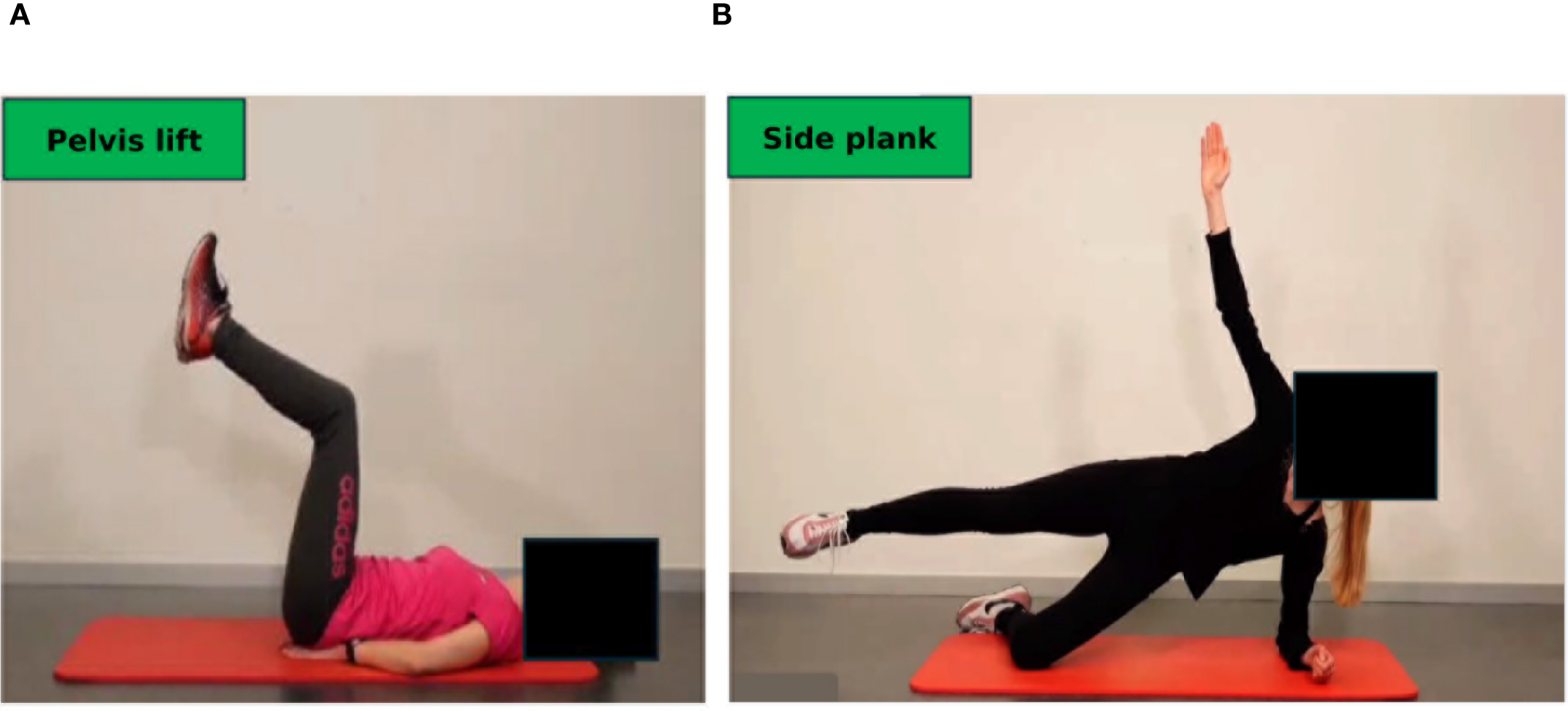
Online compendium of exercise for home-based training in the maintenance phase. Each exercise is explained, and pitfalls of incorrect performance are illustrated. **(A)** Dynamic exercise, pelvis lift: The pelvis is lifted slowly to strengthen the abdominal muscles. **(B)** Isometric exercise, side plank: The upper body must be stabilized. All exercises are available in three degrees of severity. Permission obtained from participants.

The periodization process to re-assess performance is applied before prehabilitation, during neoadjuvant and adjuvant treatment and, dependent on the patients´ risk profile, during long-term follow-up to ensure optimal benefits (18). Though initial sports consultations are usually required in an inpatient setting, outpatient services are provided in the form of on-site training guided by a sports scientist, telemedicine-based, supervised exercise training, and provision of exercise samples deposited at our website (**Figure 4**). The latter should serve as a compendium for patients to recapitulate exercises at home. Explanations are provided for patients on the correct movement execution and the aim of the exercise (educational aspect). The transition from supervised, on-site training to achieve self-directed training at home is a prerequisite to attain long-term adherence to exercise training and is facilitated by our approach. We included app-based training models to support home-based training in our cancer patients. Ahead of us there is an urgent need to further integrate remote (40) and app-based (41) training models into cancer care.

**Figure 5:**
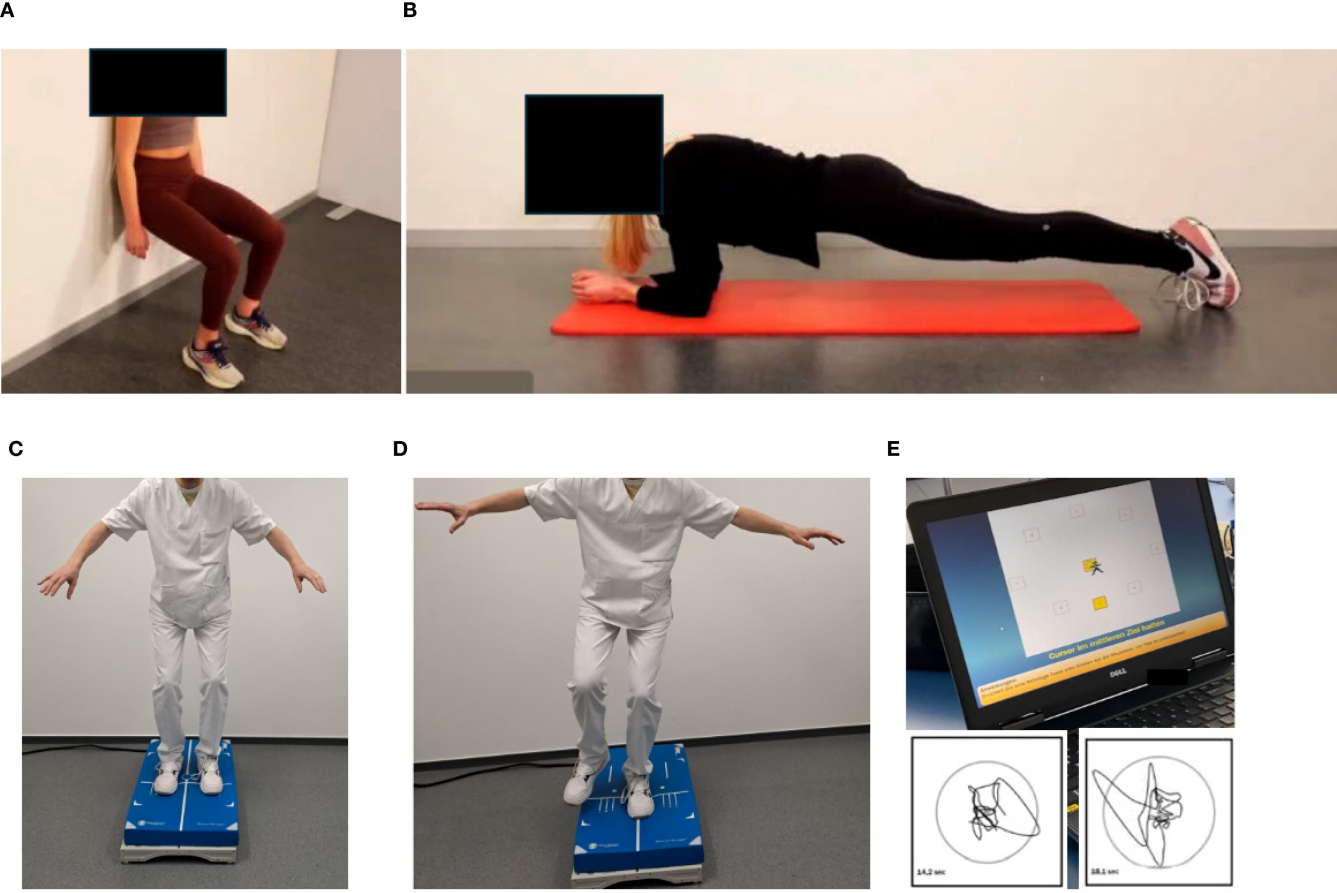
Testing of isometric strength and balance. Isometric strength is tested by holding a position as long as possible: **(A)** Squat position, **(B)** plank position. Balance is assessed by measuring two-legged **(C)** and one-legged **(D)** movement excursion, which can be graphically and numerically displayed **(E)**. Permission obtained from participants.

After clearance from the sports cardiologist repetitive testing is performed for endurance, strength and balance capacities. While endurance capacities are tested with bicycle-based CPET according to current guidelines (42–44), strength capacities are tested with machines for the main upper and lower body muscle groups. In addition, isometric tests, such as “planks” and the “squat position” are used to measure the time to uphold a technically correct position (**Figures 5A, B**). In more compromised frail patients, the Short Physical Performance Battery and the Timed Up & Go tests are applied as they have shown to correlate with survival in cancer patients (45).

Balance capacities are tested on a balance board (two-legged and one-legged, **Figures 5C, D**), movement excursions can be measured directly (**Figure 5E**).

Monitoring of heart rate (e.g. with smart watches) during the training process is desirable. Exercise training in all modalities is implemented in a stepwise approach. The concept of “start low and go slow” should be applied for all modes of training. Each training session contains a warm-up and cool down phase and features components of balance, endurance and strength exercises. Each session should start with:

1. A short coordination, vibration and balance block, which should help to antagonize fatigue, osteopenia, polyneuropathic symptoms and foster neuromuscular coordination (46, 47).
2. Endurance training is added: At the beginning of a chemo cycle intensity is low and should clearly be within the aerobic exercise intensity corridor (elected around the first ventilatory threshold (VT1) from CPET), duration should be short e.g. 5–10 min per session, increases of exercise intensity should, depending on the patient`s response, be steered through increasing frequency (e.g. two short sessions of walking/day with 5 minutes each, rather than 10 minutes once/day). As a chemo cycle progresses, both intensity (up or even above the second ventilatory threshold (VT2)) and duration e.g. 15 min per day may be increased, if lower intensities have been tolerated well by the patient. Importantly, variation should be applied in every session using elements of moderate continuous training as well as adapted (short intervals) higher intensity interval training of up to 80% of maximal exercise capacity (“adapted HIIT”). The typical “Norwegian model” of HIIT (48) (4 minute intervals at >90% maximal exercise capacity) proposed in heart failure patients does not seem to be suitable for cancer patients during chemotherapy as systemic lactate clearance may be severely compromised during this period, which may hinder active recovery (49). Thus, adapted HIIT with short intervals of 30–60 seconds (and up to two minutes of recovery) at slightly higher exercise intensities than moderate intensities may be more suitable (50).
3. Resistance training needs to be integrated from the very beginning to counteract sarcopenia, osteopenia, incontinence, and improve muscular impairment. Resistance training, when performed on machines, is mostly performed in a supervised setting. Initiation of training needs to focus on adequate technical performance to reduce the risk of injuries by using elastic bands and machines focusing on a small set of involved working muscles (50-60% of the one-repetition maximum, 1 RPM, up to 20 repetitions). The 1 RPM is usually estimated since direct measurement of maximal load may not be feasible in all facilities. In a second step, intensity can be increased reaching 70-80% of 1 RPM at 10–12 repetitions. With increasing exercise experience, dumbbells may be included to better stimulate inter- and intramuscular coordination. Integration of exercises using kinematic chains may follow in experienced cancer patients. Most importantly, resistance exercises may also be performed home-based daily including exercises with small weights or own body load e.g. squats without or with small weights.

### 2.4 Maintenance phase

Maintenance training is supported by the oncological clinics and the sports cardiological department at our university. Sports scientists are employed to ensure long-term adherence to exercise by offering exercise counseling for cancer patients. This includes advice on outpatient contacts to physiotherapists specialized in cancer patients as well as education on the role of exercise to treat cancer-related side effects, such as incontinence, fatigue, polyneuropathy, obstipation and depression (sports scientific consultation hour). In- and outpatient exercise training is offered on site by sports scientists. Moreover, the university hospital program is closely connected to physiotherapy facilities within the greater urban area with knowledge and expertise in specifically treating cancer patients in different outpatient phases. Furthermore, patients are educated to consult our website to recapitulate the exercises on balance and flexibility, cardio and resistance training (www.sport.mri.tum.de) and provide a smooth transfer from supervised on-site to home-based self-guided training. This training can be supported by telemedicine-based, supervised training. In our experience, referring to the opportunity of supported training at home early in the treatment process, fosters self-determined exercise and improves long-term adherence.

## 3 Discussion

We present a novel approach of repetitive cardiopulmonary exercise stress testing of the entire oxygen cascade and analysis of the determinants of VO_2peak_ to unmask individual exercise limitations. We thus transfer a sport scientific periodization model to cancer patients. Our approach is new in several ways (1): Our interdisciplinary approach with feedback of exercise testing results to the treating surgeons, medical and radiation oncologists could facilitate adaptations of treatment regimens to prevent CTRCD, cardiotoxicity and heart failure. (2) Early signs of cardiotoxicity, CTRCD or heart failure may only become visible during exercise testing. By detecting early signs of dysfunction, adaptation of cancer therapy regimens may further reduce the risk of adverse events on top of optimized medical and radiation treatment. (3) In a time of precision medicine and oncology our exercise testing protocol of the entire oxygen cascade provides a tool to create a “cardiopulmonary exercise signature” of each cancer patient. As cancers with different molecular signatures may respond differently to exercise stimuli, exercise prescriptions need to be based on these findings. Resistance training is an effective tool to improve arteriovenous oxygen difference (37, 51), which is often hampered in cancer patients (52, 53). Detection of this limitation can result in focusing exercise prescriptions on resistance training. On the other hand, failure to improve stroke volume and heart rate reserve (central limitations) during simultaneous stress echocardiography and CPET testing may require further cardiological assessment to unmask underlying structural heart disease or cardiovascular disease. In addition, exercise prescriptions to improve central limitations could include a higher amount of endurance training using elements of moderate-continuous, but also short bouts of high intensity training (18, 54). In summary, we present a sports cardio-oncological approach of precision medicine to prescribe exercise based on the individual exercise response of the entire cardiopulmonary system, rather than endurance exercise prescriptions based only on VO_2peak_. Our approach should be implemented into future exercise oncology studies to delineate whether different cancer entities and molecular signatures require specific exercise interventions.

Our approach highlights the need for early involvement of cardiologists in high risk patients, as suggested by current cardio-oncological guidelines (27), but extends current suggestions from resting echocardiography and cardiac biomarkers to patient assessment during stress conditions. Both CPET and stress echocardiography are feasible tools in daily sports cardiological routine and bear great potential to be implemented into exercise oncology. In summary, our approach provides novel aspects of exercise prescriptions, which are continuously adapted and reported to the treating physicians. Using this “exercise signature” of the entire oxygen cascade in cancer patients to steer exercise prescriptions is novel and requires further studies to achieve a reduction in cardiotoxicity, CTRCD and heart failure.

### 3.1 Next research steps

Our approach is currently being tested in a single-center, randomized controlled exercise intervention trial in triple negative breast cancer patients undergoing neoadjuvant anthracycline-based and immune checkpoint inhibitor therapy (Exercise in regional breast cancer with neoadjuvant Anthracycline-based ChemoTherapy with Immune Checkpoint-Inhibition (ExACT-ICI): A prospective randomized controlled trial, NCT06672120) (55). The ExACT-ICI trial is a prospective single-center study randomizing (1:1) 120 TNBC patients (stage I-III, age 18-65) before initiating anthracycline (AC)-based chemotherapy plus pembrolizumab into 1.) Exercise Training (ET) or 2.) Usual Care. ET participants will perform video-supervised sessions (endurance, resistance, balance) 5x/week during 24-week treatment, monitored via smartwatch. Both groups will undergo detailed cardiological assessment (combined cardiopulmonary exercise testing and stress echocardiography) and strength/balance assessments at baseline, 6, 12, and 24 weeks. The primary outcome is VO_2peak_ change from baseline to 24 weeks (a difference of 2mL/kg/min is deemed significant, power 90%), with secondary outcomes including changes of cardiac biomarkers, echocardiographic variables, quality of life, strength, balance and adverse cardiac events (incidence of cardiotoxicity, immune checkpoint associated myocarditis, CTRCD, cardiovascular events, atrial and ventricular arrhythmias, and heart failure hospitalizations) through follow-up of 52 weeks. Eligibility for study inclusion is assessed in our interdisciplinary board. Exercise prescriptions and follow-up adhere to our proposal depicted in this manuscript. We hope that this study will help to further establish our approach in sports cardio-oncological care and can be extended to other cancer entities.

### 3.2 Challenges and limitations of exercise oncology in clinical routine

Costs are a considerable limitation in exercise oncology. Our approach using CPET and stress echocardiography is feasible in our facility due to our experience in performing high-volume exercise testing (37, 51, 56). We admit that this may not be reproducible in other centers. As the role of exercise as a cancer drug to improve mortality has been demonstrated (9), contracts with insurance companies need to be made to lower costs. In addition, detailed exercise prescriptions may be restricted to cancer patients at high risk for adverse events, which can be assessed by the validated HFA-ICOS score (27, 57) prior to treatment initiation. Our approach to provide a transition from supervised on-site to home-based supervision should increase adherence to long-term exercise, also supported by provision of exercises on demand for self-directed training (www.sport.mri.tum.de). It needs to be considered though, that home-based training may not be applicable to all cancer entities, especially those with poor prognosis or a high number of comorbidities. Thus, it will remain at the discretion of the sports cardiologist to select the most appropriate training approach for each patient.

## 4 Conclusions

We present an interdisciplinary periodization approach as a potential role model for long-term cancer care which could be implemented on a larger scale following studies demonstrating a further reduction of CTRCD, cardiotoxicity and heart failure through exercise on top of state-of-the-art cancer therapy. Tailoring repetitive exercise prescriptions based on impairments in VO_2peak_ determinants is key to success and should be part of precision sports cardio-oncology. This can be achieved by implementation of simultaneous CPET and stress echocardiography, a clinically feasible assessment to analyze the entire oxygen cascade during exercise. We highlight that early education on self-determined exercise is key in a time of short financial resources and limited availability of cancer sports groups and physiotherapists. Ensuring a smooth transition from in-hospital, supervised training to home-based, remote and app-supported training will be the future of exercise training in cancer. Judging from the undoubted beneficial effect of exercise the time may be ripe to implement the concept of a *SportsCardioOncology Board* into the oncological routine to better adapt exercise, medical, surgical and radiation treatment regimens over time as part of the precision medicine concept and further improve survival of cancer patients.

## Data Availability

The original contributions presented in the study are included in the article/Supplementary Material. Further inquiries can be directed to the corresponding author.
